# Relationship between enteral nutrition and serum levels of inflammatory factors and cardiac function in elderly patients with heart failure: A protocol for systematic review and meta-analysis

**DOI:** 10.1097/MD.0000000000025891

**Published:** 2021-05-14

**Authors:** Shutang Zhao, Xiren Wang

**Affiliations:** a Department of Cardiology, Weihaiwei People's Hospital; b Department of Laboratory, Weihai Municipal Hospital, Shandong, China.

**Keywords:** cardiac function, enteral nutrition, heart failure, inflammatory factors

## Abstract

**Background:**

: Published studies investigating enteral nutrition's effect on serum inflammatory factors and the cardiac function of malnourished elderly patients with heart failure (HF) are of poor quality, with small sample sizes, and involve a homogeneous population. Therefore, in order to provide new medical evidence for clinical treatment, we undertook a systematic review and meta-analysis to assess the relationship between enteral nutrition and serum levels of inflammatory factors and cardiac function in elderly patients with HF.

**Methods:**

: The protocol was written following the Preferred Reporting Items for Systematic Reviews and Meta-Analyses Protocols (PRISMA-P) statement guidelines. Electronic databases including Web of Science, Embase, PubMed, Wanfang, Data, Scopus, Science Direct, Cochrane Library will be searched in April 2021 by 2 independent reviewers. The primary outcome is body mass index, triceps skin fold thickness, upper arm muscle circumference, serum total protein, albumin, and hemoglobin's change in index; secondary outcomes include left ventricular ejection fraction, B-type natriuretic peptide, interleukin-6, C-reactive protein, and tumor necrosis factor-α. The risk of bias assessment of the included studies was performed by 2 authors independently using the tool recommended in the Cochrane Handbook for Systematic Reviews of Interventions (version 5.1.0). We will perform meta-analysis using Review Manager Software.

**Results:**

: The review will add to the existing literature by showing compelling evidence and improved guidance in clinic settings.

**Conclusion:**

: Its findings will provide helpful evidence for the application of enteral nutrition in elderly patients with HF.

**OSF registration number:**

10.17605/OSF.IO/RTYBP.

## 1 Introduction

Heart failure (HF) is a clinical syndrome characterized by impaired cardiac function due to various cardiovascular diseases and has a mortality of approximately 50%.^[1,2]^ Although progress has been achieved in HF treatment in recent years, the 5-year mortality of patients with HF remains high. HF can be caused by diseases of the endocardium, myocardium, pericardium, heart valves, and vessels or by metabolic disorders.^[3,4]^ Most patients with HF have symptoms related to impaired left ventricular myocardial function. Patients usually present with dyspnea, fatigue limiting exercise tolerance, and fluid retention characterized by pulmonary and peripheral edema.

The use of diuretics, β-blockers, angiotensin-converting enzyme inhibitors, angiotensin receptor blockers, angiotensin receptor neprilysin inhibitor, hydralazine plus nitrate, digoxin, and aldosterone antagonists can relieve symptoms.^[5,6]^ Prolongation of patient survival has been documented after the use of β-blockers, angiotensin-converting enzyme inhibitors, angiotensin receptor neprilysin inhibitor, hydralazine plus nitrate, and aldosterone antagonists. More limited evidence of survival benefit is available for diuretic therapy. The angiotensin-converting enzyme inhibitors or angiotensin receptor blockers were replaced by angiotensin receptor neprilysin inhibitor in the treatment of chronic symptomatic patients with chronic heart failure New York Heart Association class II–III heart failure^[7,8]^ and adequate blood pressure who could tolerate an optimal dose of these medications. Angiotensin receptor neprilysin inhibitor should not be given within 36 hours after angiotensin-converting enzyme inhibitors dose.

A failing heart is similar to an engine running out of energy. Regulating cardiac energy metabolism is expected to become a new method for the treatment of HF.^[9,10]^ Currently, there is limited report on the utilization of energy metabolism to treat HF. We performed this protocol for systematic review and meta-analysis to investigate enteral nutrition's effect on serum inflammatory factors and the cardiac function of malnourished elderly patients with HF.

## 2 Methods

### 2.1 Protocol registration

The prospective registration has been approved by the Open Science Framework registries, and the registration number is 10.17605/OSF.IO/RTYBP. The protocol was written following the Preferred Reporting Items for Systematic Reviews and Meta-Analyses Protocols (PRISMA-P) statement guidelines.^[11]^ Ethical approval and patient consent are not required because this study is a literature-based study.

### 2.2 Searching strategy

Electronic databases including Web of Science, Embase, PubMed, Wanfang, Data, Scopus, Science Direct, Cochrane Library will be searched in April 2021 by 2 independent reviewers. For search on PubMed, the following search terms will be used: “enteral nutrition,” “heart failure,” “cardiac function”. To minimize the risk of publication bias, we will conduct a comprehensive search that included strategies to find published and unpublished studies. The reference lists of the included studies will also be checked for additional studies that are not identified with the database search. There is no restriction in the date of publication or language in the search.

### 2.3 Eligibility criteria

Studies included in this review have to meet all of the following inclusion criteria in the PICOS order:

1. population: patients with HF;
2. intervention group (group A): 500 mL d^−1^ of enteral nutrition for 1 month;
3. comparison group (group B): free diet;
4. outcome measures: the primary outcome is body mass index, triceps skin fold thickness, upper arm muscle circumference, serum total protein, albumin, and hemoglobin's change in index; secondary outcomes include left ventricular ejection fraction, B-type natriuretic peptide, interleukin-6, C-reactive protein, and tumor necrosis factor-α;
5. study design: randomized controlled trial.

Biomechanical studies, non-randomized cohort studies, in vitro studies, review articles, techniques, case reports, letters to the editor, and editorials are excluded.

### 2.4 Study selection

The first author will conduct a preliminary screening based on the title to eliminate any research not related to the topic. A log of excluded studies is kept with the rationale for exclusion. Subsequently, all remaining abstracts will be reviewed by the primary author, and the selection criteria are applied. Studies identified for full text review will be evaluated by 2 authors for inclusion in the study. Disagreements will be resolved through a discussion with a third review author. Journal titles and authors’ names will be not glossed over in the research selection process. A manual search of the bibliographies of included studies is performed to ensure that the overall search was comprehensive and complete.

### 2.5 Data extraction

Two independent authors will extract the following descriptive raw information from the selected studies: study characteristics such as author, study design, study language, publication year, mean follow-up period; patient demographic details such as number, average age, body mass index and gender ratio; details of interventions, and outcome measures. If the data are missing or can not be extracted directly, we will contact the corresponding authors to ensure that the information integrated. Otherwise, we will calculate them with the guideline of Cochrane Handbook for Systematic Reviews of Interventions 5.1.0. If necessary, we will abandon the extraction of incomplete data.

### 2.6 Quality assessment

The risk of bias assessment of the included studies was performed by 2 authors independently using the tool recommended in the Cochrane Handbook for Systematic Reviews of Interventions (version 5.1.0). This tool included 7 aspects which were sequence generation (selection bias), allocation sequence concealment (selection bias), blinding of participants and personnel (performance bias), blinding of outcome assessment (detection bias), incomplete outcome data (attrition bias), selective outcome reporting (reporting bias), and other bias (baseline balance and fund). Additionally, each of the aspects was ranked low risk of bias, high risk of bias, and unclear risk of bias.

### 2.7 Statistical analysis

We will perform meta-analysis using Review Manager Software (RevMan Version 5.4, The Cochrane Collaboration, Copenhagen, Denmark). If available data are insufficient, a descriptive analysis will be carried out. The Q-test and *I*^2^ values will be used to indicate inter-study heterogeneity. When the *P* value of Q-test >.1 and *I*^2^ < 50%, a fixed-effects model was applied; otherwise, a random-effects model was used. Binary variables were expressed by odds ratio with 95% confidence interval, and continuous variables by mean difference with 95% confidence interval. If significant heterogeneity is found, we will try to explore the source of heterogeneity by subgroup analysis based on specified effect modifiers as follows: interventions, publication year, participant's average age, sample size, publication language, and so on.

## 3 Discussion

Myocardial energy metabolism influences not only its systolic function, but also its diastolic function.^[12,13]^ If the body cannot produce enough energy, it causes operating problems in the heart, which lead to heart diseases. Elderly patients with HF usually have congested system circulation or pulmonary circulation featured by long course of disease, bad nutritional status, and substantial weight loss, which easily aggravates heart energy metabolism and leads to HF.^[14]^ Enteral nutrition is an intestinal immune compound preparation. It is a polymer readily metabolized with reasonable combination and designed in accordance with human metabolic characteristics. Published studies investigating enteral nutrition's effect on serum inflammatory factors and the cardiac function of malnourished elderly patients with HF are of poor quality, with small sample sizes, and involve a homogeneous population. Therefore, in order to provide new medical evidence for clinical treatment, we undertook a systematic review and meta-analysis to assess the relationship between enteral nutrition and serum levels of inflammatory factors and cardiac function in elderly patients with HF.

## Author contributions

**Conceptualization**: Shutang Zhao.

**Data curation**: Xiren Wang.

**Investigation:** Xiren Wang.

**Methodology:** Shutang Zhao.

**Resources:** Xiren Wang.

**Software:** Shutang Zhao.

**Supervision:** Shutang Zhao.

**Writing – original draft:** Shutang Zhao.

**Writing – review & editing:** Xiren Wang.
